# Scottish Local Authorities and Sanatorium Treatment

**Published:** 1921-02-05

**Authors:** 


### Scottish Local Authorities and Sanatorium Treatment.

There appears to be some misunderstanding of the position of sanatorium benefit in Scotland. In England sanatorium benefit will not cease until May 1, to be included among the benefits conferred upon insured persons by Part I. of the National Insurance Act, 1911; but in Scotland this termination has actually taken place, and as from January 1, 1921, the Public Health Local Authorities have been required to make arrangements for the treatment of tuberculosis in insured persons. A special scheme under which treatment will be afforded by local authorities in Scotland to ex-Service men suffering from tuberculosis has been devised, so that there shall be no break in the continuity of the treatment. The local authorities are to receive in respect of the treatment of insured persons suffering from tuberculosis an Exchequer grant, which may be taken broadly as representing the amount that has been available to local Insurance Committees for institutional treatment; and in respect of the treatment of ex-Service men the local authorities will obtain reimbursement of the entire cost of institutional treatment, and will also receive payment for the services of their tuberculosis officers.

